# New Interventions for Older Adult Victim-Survivors of Financial Fraud

**DOI:** 10.1093/geroni/igaf122.655

**Published:** 2025-12-31

**Authors:** Marguerite DeLiema, Surya Kolluri

## Abstract

Financial fraud is a growing elder justice issue. While people of all ages are targeted, isolated older adults without a financial or social safety net experience the most significant harm. Victimization often comes with deep shame, making it hard to reach out for help or even acknowledge the consequences. There is also very little research on what works to protect older adults from fraud victimization. This symposium will bring together researchers and direct practice clinicians to discuss findings from their recent work to prevent fraud and intervene to support victim-survivors. The first presentation will showcase “Successful Aging thru Financial Empowerment” (SAFE), a no cost financial coaching and economic advocacy service dedicated to preventing financial exploitation and fraud among older adults. The program also provides financial and persuasion literacy training and scam awareness education. SAFE Director, Latoya Hall, MSW, will present research findings on a pilot study and longitudinal findings on financial fraud victims who received SAFE services. Next, Marti DeLiema, PhD, will share research on the efficacy of temporary financial account holds as a stopgap measure to protect older adults from continued financial exploitation, and also the impact these holds have on victim-survivors’ financial autonomy. Last, practitioners with FightCybercrime will present data on how online peer support groups for romance fraud victim-survivors assist with emotional recovery and help build community following fraud. The presentations will be followed by a panel discussion led by Surya Kolluri (Director, TIAA Institute) on the future of fraud intervention research.

